# Building Natural Product Libraries Using Quantitative Clade-Based and Chemical Clustering Strategies

**DOI:** 10.1128/mSystems.00644-21

**Published:** 2021-10-26

**Authors:** Victoria M. Anderson, Karen L. Wendt, Fares Z. Najar, Laura-Isobel McCall, Robert H. Cichewicz

**Affiliations:** a Natural Products Discovery Group, University of Oklahoma, Norman, Oklahoma, USA; b Institute for Natural Products Applications and Research Technologies, University of Oklahoma, Norman, Oklahoma, USA; c Department of Chemistry and Biochemistry, University of Oklahoma, Norman, Oklahoma, USA; d Chemistry and Biochemistry Bioinformatics Core, University of Oklahoma, Norman, Oklahoma, USA; e Department of Microbiology and Plant Biology, University of Oklahoma, Norman, Oklahoma, USA; f Laboratories of Molecular Anthropology and Microbiome Research, University of Oklahoma, Norman, Oklahoma, USA; INDICASAT

**Keywords:** natural products, LC-MS metabolomics, chemical diversity, drug discovery, fungi, library design, metabolomics

## Abstract

The success of natural product-based drug discovery is predicated on having chemical collections that offer broad coverage of metabolite diversity. We propose a simple set of tools combining genetic barcoding and metabolomics to help investigators build natural product libraries aimed at achieving predetermined levels of chemical coverage. It was found that such tools aided in identifying overlooked pockets of chemical diversity within taxa, which could be useful for refocusing collection strategies. We have used fungal isolates identified as *Alternaria* from a citizen-science-based soil collection to demonstrate the application of these tools for assessing and carrying out predictive measurements of chemical diversity in a natural product collection. Within *Alternaria*, different subclades were found to contain nonequivalent levels of chemical diversity. It was also determined that a surprisingly modest number of isolates (195 isolates) was sufficient to afford nearly 99% of *Alternaria* chemical features in the data set. However, this result must be considered in the context that 17.9% of chemical features appeared in single isolates, suggesting that fungi like *Alternaria* might be engaged in an ongoing process of actively exploring nature’s metabolic landscape. Our results demonstrate that combining modest investments in securing internal transcribed spacer (ITS)-based sequence information (i.e., establishing gene-based clades) with data from liquid chromatography-mass spectrometry (i.e., generating feature accumulation curves) offers a useful route to obtaining actionable insights into chemical diversity coverage trends in a natural product library. It is anticipated that these outcomes could be used to improve opportunities for accessing bioactive molecules that serve as the cornerstone of natural product-based drug discovery.

**IMPORTANCE** Natural product drug discovery efforts rely on libraries of organisms to provide access to diverse pools of compounds. Actionable strategies to rationally maximize chemical diversity, rather than relying on serendipity, can add value to such efforts. Readily implementable biological (i.e., ITS sequence analysis) and chemical (i.e., mass spectrometry-based feature and scaffold measurements) diversity assessment tools can be employed to monitor and adjust library development tactics in real time. In summary, metabolomics-driven technologies and simple gene-based specimen barcoding approaches have broad applicability to building chemically diverse natural product libraries.

## INTRODUCTION

Drug discovery has changed tremendously during the last century, with the process undergoing continuous reinvention to avail itself of new scientific methods and trends. Numerous ideas and tools have been put into practice, resulting in the creation of many chemical collections used in modern drug screening and molecular probe development throughout academia, industry, and government. Small-molecule libraries based on organic compounds of various sizes (e.g., <900 Da for most synthetic libraries but ranging up to around ∼2,000 Da for some natural product collections) play a dominant role in such efforts, with many collections accommodating a variety of screening and discovery methodologies (e.g., fragment based, target focused, diversity oriented, combinatorial, DNA encoded, repurposed, and virtual) (1–6).

Despite the vast amounts of time, money, and energy poured into building small-molecule screening collections, the answers to many basic questions about their design and development, such as optimal collection sizes, are largely driven by adherence to dogma or convenience rather than evidence-based reasoning. Such questions grow increasingly relevant, as opinions influencing the last 4 decades of library design have shifted tremendously, with the large collections of the 1980s and 1990s (e.g., combinatorial chemistry [7]) being replaced by smaller tailored collections in the early 2000s (e.g., “focused” collections [8, 9]) and moving toward megascale libraries in recent years (e.g., DNA encoded libraries [10–15]).

While such trends are strongly linked to the creation of synthetic chemical collections, a similar set of concerns applies to the construction of libraries assembled from natural sources (e.g., microorganisms and plants). Many ideas have emerged related to best practices for building natural product libraries, with extracts, fractions, and pure compounds defining the three dominant types of chemical complexity encountered in screening collections (16–19). Despite the tremendous ingenuity and effort that have gone into assessing these and other methods of building natural product libraries, comparatively less consideration has been given to identifying optimal sample sizes needed to construct nature-based screening collections. Answering such questions is important since the degree of chemical diversity in a screening collection is considered a key contributor to the success (or failure) of bioassay screening endeavors (20, 21).

A possible reason for neglecting this problem may stem from the fact that as opposed to synthetic libraries, natural products are encountered not as single molecules but as compound sets (e.g., metabolomes) representing the total metabolic output of each organism. Given the degree to which natural product biosynthetic gene clusters and their molecular controlling factors are swapped, recombined, and otherwise altered within host organisms, even the metabolomes of low-ranking monophyletic clades (e.g., a species or genera) can exhibit divergent chemical profiles (22, 23). These factors can make the rational design of natural product libraries challenging. Therefore, methods to perform chemical diversity measurements have the potential to aid and inform the design of natural product drug screening collections.

Two examples help illustrate the practical need for solving this problem. In an intriguing opinion piece offered by Baltz, various scenarios were offered to overcome the global slowing of antibiotic discovery from actinomycetes (order: *Actinomycetales* Buchanan, 1917) (24). Based on that analysis, it was concluded that using traditional bioassay-guided antibacterial discovery alone would require testing >10^7^ actinomycetes to identify the next, major new class of antibiotic. Although this estimate was highly theoretical and based on the use of standard bioassay-driven screening procedures, it provided a compelling starting point for considering how the integration of compound diversity measurements into bioassay screening could help serve as a chemically focused approach to assessing real and presumed barriers to natural product discovery. In another case, Letzel and colleagues carried out a survey of natural product biosynthetic gene cluster diversity represented in 119 *Salinispora* sp. genomes (25). A key takeaway from the study was that despite high levels of global gene conservation among *Salinispora* isolates, roughly half of all the biosynthetic gene clusters detected were found in two or fewer isolates. Thus, deep sampling of this genus was expected to continue yielding new families of natural products. With no end in sight for the sustained emergence of new natural products (26), questions surrounding how to define, measure, and construct optimally sized natural product-based chemical libraries take on critical importance.

Fungi epitomize many of the challenges inherent in sourcing natural products and thus serve as a useful starting point for establishing a quantitative approach to natural product library design. Topmost among the difficulties working with fungi are the complex, and in many cases poorly resolved, taxonomic relationships exhibited by these organisms. For example, many fungi adopt different sexual states that are metabolically and morphologically distinct. Historically, such cases have resulted in fungal isolates that exhibit gene-level equivalencies being assigned different binomial names (27). In other instances, the high degree of genetic diversity within certain fungal clades has created taxonomic quagmires that have left some fungi loosely classified into poorly defined species complexes, polyphyletic clades, and paraphyletic groups (28, 29). Complicating these matters, the regional variation and global distribution of most fungal taxa remain poorly defined, which has given rise to unresolved questions about the true extent of biological and chemical diversity throughout the fungal kingdom. Here, we present a set of guiding principles for combining, quantifying, and assessing chemical and source organism diversity during the construction of natural product libraries. Our efforts focused on *Alternaria* Ness, which is a cosmopolitan and taxonomically perplexing fungal genus (30, 31) known to produce many types of metabolites (32–37). Although these experiments concentrated on fungi, we expect that the procedures laid out here will be generally applicable to the evaluation of natural products from other source organisms.

## RESULTS AND DISCUSSION

### Basis for a bifunctional analysis tool to assess *Alternaria* ITS barcode and chemical diversity.

The *Alternaria* isolates used in this study were obtained through the University of Oklahoma, Citizen Science Soil Collection Program (38, 39), which to date has received 9,670 soil samples from across the United States, yielding 78,581 fungal isolates identified by single-read internal transcribed spacer (ITS) sequencing data. A query performed on the ITS barcode data yielded an initial set of 219 candidate *Alternaria* isolates, which was refined to a subset of 198 samples having >90% ITS sequence similarity (40–42) to *Alternaria* type strain data available in GenBank and defined by Woudenberg et al. (31). Upon plating, all strains exhibited colony morphologies consistent with the genus *sensu stricto*.

*Alternaria* exemplifies many of the practical problems and limitations that researchers face when developing natural product libraries. Specifically, *Alternaria* is a taxon in flux, having undergone revisions as mycologists have striven to consider morphological characteristics, telemorphic states, various marker genes, and more to delineate this group and its allied genera (28, 31, 43). While the outcomes of those efforts have differed, resulting in proposals supporting various combinations of monophyletic species groups and species complexes, they have found agreement on the grounds that *Alternaria* exhibits tremendous morphological and genetic plasticity. Recognizing that these problems are common throughout the microbial world, we adopted a hybrid method of library construction focused on assessing the prospective taxonomic affinity of each isolate (preferably to a genus-level taxon using ITS barcode sequence results) in combination with liquid chromatography-mass spectrometry (LC-MS) metabolome profiling data. This bifunctional approach offered insights into the relationship between phylogeny and chemistry, which enabled (i) assessment of natural product chemical diversity within species complexes, (ii) identification of prospective pools of under- and oversampled secondary-metabolite scaffolds, and (iii) application of quantitative metrics to establish and track goals concerning chemical diversity in an existing or growing natural product collection. Whereas numerous tactics have been reported for guiding natural product library development (44–46), we view our approach as a departure from prior schemes, considering its quantitative aspects that we now explore.

### Characterizing ITS barcode (clades) and metabolome (clusters) based groups in *Alternaria*.

While achieving a state of perfect knowledge about the evolutionally histories of microorganisms is nearly impossible, we can use certain low-cost and minimally intensive tools to gain functional insights concerning their phylogenetic relationships. For fungi, the ITS barcode system serves as one such tool, offering an efficient way to establish a working set of phylogenetic associations among environmental isolates (29). The phylogenetic analysis of *Alternaria* ITS data revealed five sequence-based clades (clades U, V, W, X, and Y). Whereas further taxonomic resolution might be achievable using additional genetic markers, ITS provides a reasonable method to identify isolates and draw attention to potential points of evolutionary divergence (27, 29).

Principal-coordinate analysis (PCoA) was performed on the *Alternaria* metabolomics data. The components detected in *Alternaria* metabolomes were treated as chemical features based on a combination of their LC retention times and mass-to-charge ratio. Those efforts resulted in a model that supported the presence of six chemical clusters (clusters 1, 2, 3, 4, 5, and 6) among the *Alternaria* isolates (see Fig. S1 in the supplemental material).

**FIG 1:**
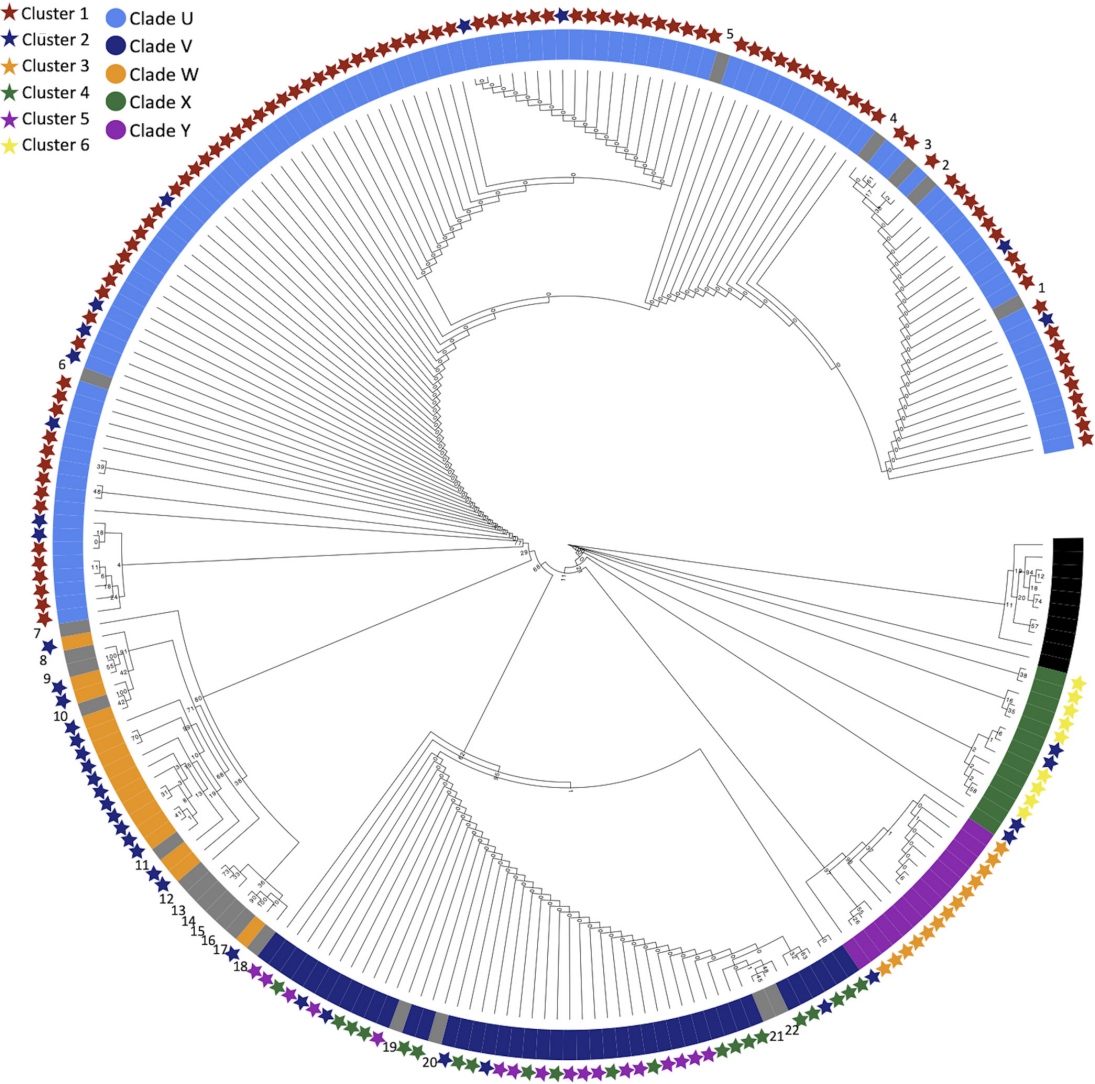
Genetic and chemical clustering of *Alternaria*. ITS phylogeny of *Alternaria* isolates is shown. Inner ring indicates the clade, while color-coded stars represent the chemical cluster. The clades and clusters show remarkable overlap but also reveal a hidden chemical cluster within clade V. Numbers indicate type strain data from GenBank (Table S2).

10.1128/mSystems.00644-21.1 FIG S1 Principal-coordinate analysis and hierarchical clustering of *Alternaria*. Chemical cluster is indicated by colors, which appear in Fig. 1 in the main text (permutational multivariate analysis of variance [PERMANOVA], *P* = 0.001 by chemical cluster). Download FIG S1, TIF file, 0.8 MB. Copyright © 2021 Anderson et al. 2021 Anderson et al. https://creativecommons.org/licenses/by/4.0/ This content is distributed under the terms of the Creative Commons Attribution 4.0 International license.

The results generated from the ITS barcode and metabolomics data sets were overlaid, demonstrating a high degree of consensus between the two models (Fig. 1). The data indicated that clade U was composed primarily of chemical cluster 1, clade W was composed of chemical cluster 2, clade X was composed primarily of chemical cluster 6, and clade Y was composed of chemical cluster 3. Notably, clade V contained both clusters 4 and 5. This underscored the value of layering chemical data (clusters) on top of genetic data (clades) to reveal otherwise unexpected pockets of chemical divergence within genetic groups. A few cases were noted in the principal-coordinate analysis, revealing that some members of chemical cluster 2 were embedded in clades U, V, and X. Although the reasons behind these cases are uncertain, we speculate that it may be due to culture-dependent effects on metabolite production (47) and/or genomic/epigenome-scale events that resulted in the loss of chemical scaffolds (48, 49), which served to differentiate clusters 1, 3, 4, 5, and 6 from cluster 2. Analyses in this report were conducted in parallel on both clade and cluster models, with the chemical cluster model generating results similar to those of the clade model (Fig. S2B and C and Fig. S3, S5, and S7).

10.1128/mSystems.00644-21.2 FIG S2 Feature richness and diversity of *Alternaria*. (A) Feature count with random selection of isolates from larger clades (*n* = 26). Significant differences in the chemical richness of clades persisted even when the sample size was subsampled to achieve a balanced dataset (*P* < 0.001). “*” indicates statistically significant difference from clade U. “‡” indicates statistically significant difference from clade V. “Ⓧ” indicates statistically significant difference from clade W. “⋄” indicates statistically significant difference from clade Y. (B) Feature count by chemical cluster. Chemical clusters also showed significant differences in chemical richness when analyzed as a whole (*P* < 0.001). “⋄” indicates statistically significant difference from cluster 2. (C) Feature count with random selection of isolates from larger clusters (*n* = 18). Chemical richness of a balanced data set (*n* = 18) yielded significant differences between chemical clusters (*P* = 0.0338). “*” indicates statistically significant difference from cluster 1. “‡” indicates statistically significant difference from cluster 3. Download FIG S2, TIF file, 1.5 MB. Copyright © 2021 Anderson et al. 2021 Anderson et al. https://creativecommons.org/licenses/by/4.0/ This content is distributed under the terms of the Creative Commons Attribution 4.0 International license.

10.1128/mSystems.00644-21.3 FIG S3 Venn diagram of features in chemical clusters 1 to 6. Feature overlap by chemical cluster is tremendously complex. Clusters were constructed based on hierarchical clustering analysis using a Bray-Curtis distance metric. There is a high degree of overlap between these clusters: 5,166 (47.0%) features are shared by at least 2 chemical clusters. The remaining 5,825 (53.0%) features are unique to a single cluster: 2,516 (22.9%), 1,857 (16.9%), 863 (7.9%), 185 (1.7%), 217 (2%), and 187 (1.7%) of features were found to be unique to clusters 1, 2, 3, 4, 5, and 6, respectively. Download FIG S3, TIF file, 2.1 MB. Copyright © 2021 Anderson et al. 2021 Anderson et al. https://creativecommons.org/licenses/by/4.0/ This content is distributed under the terms of the Creative Commons Attribution 4.0 International license.

10.1128/mSystems.00644-21.5 FIG S5 Venn diagram of scaffolds in chemical clades 1 to 6. A total of 1,185 (71.3%) scaffolds were found to be shared between at least two chemical clusters, while the remaining 476 (28.7%) scaffolds were found to be unique to a single chemical cluster. Of these scaffolds, 197 (11.9%), 154 (9.3%), 76 (4.6%), 11 (0.7%), 21 (1.3%), and 17 (1%) were found to be unique to chemical clusters 1, 2, 3, 4, 5, and 6, respectively. Download FIG S5, TIF file, 1.9 MB. Copyright © 2021 Anderson et al. 2021 Anderson et al. https://creativecommons.org/licenses/by/4.0/ This content is distributed under the terms of the Creative Commons Attribution 4.0 International license.

10.1128/mSystems.00644-21.7 FIG S7 Exploration of scaffold-level diversity within chemical clusters. A library that was constructed exclusively of isolates from the most abundant clade (clade 1) would provide access to 74.8% of scaffolds. The addition of clusters 2, 3, 4, 5, and 6 provides an additional 16.1%, 5.5%, 1.3%, 1.3%, and 1.0%. However, if the library emphasized the smaller clusters, the 96 isolates that make up clusters 2 to 6 provide access to 87.9% of total scaffolds and the addition of cluster 1 provides only 12.1% of the total scaffolds. Download FIG S7, TIF file, 1.3 MB. Copyright © 2021 Anderson et al. 2021 Anderson et al. https://creativecommons.org/licenses/by/4.0/ This content is distributed under the terms of the Creative Commons Attribution 4.0 International license.

10.1128/mSystems.00644-21.9 TABLE S2 *Alternaria* type strains identified in GenBank that were used to create ITS-based clades. The *Alternaria* spp. are identified by number (i.e., number in tree) in the cladogram shown in Fig. 1 of the main text. Download Table S2, DOCX file, 0.01 MB. Copyright © 2021 Anderson et al. 2021 Anderson et al. https://creativecommons.org/licenses/by/4.0/ This content is distributed under the terms of the Creative Commons Attribution 4.0 International license.

**FIG 2:**
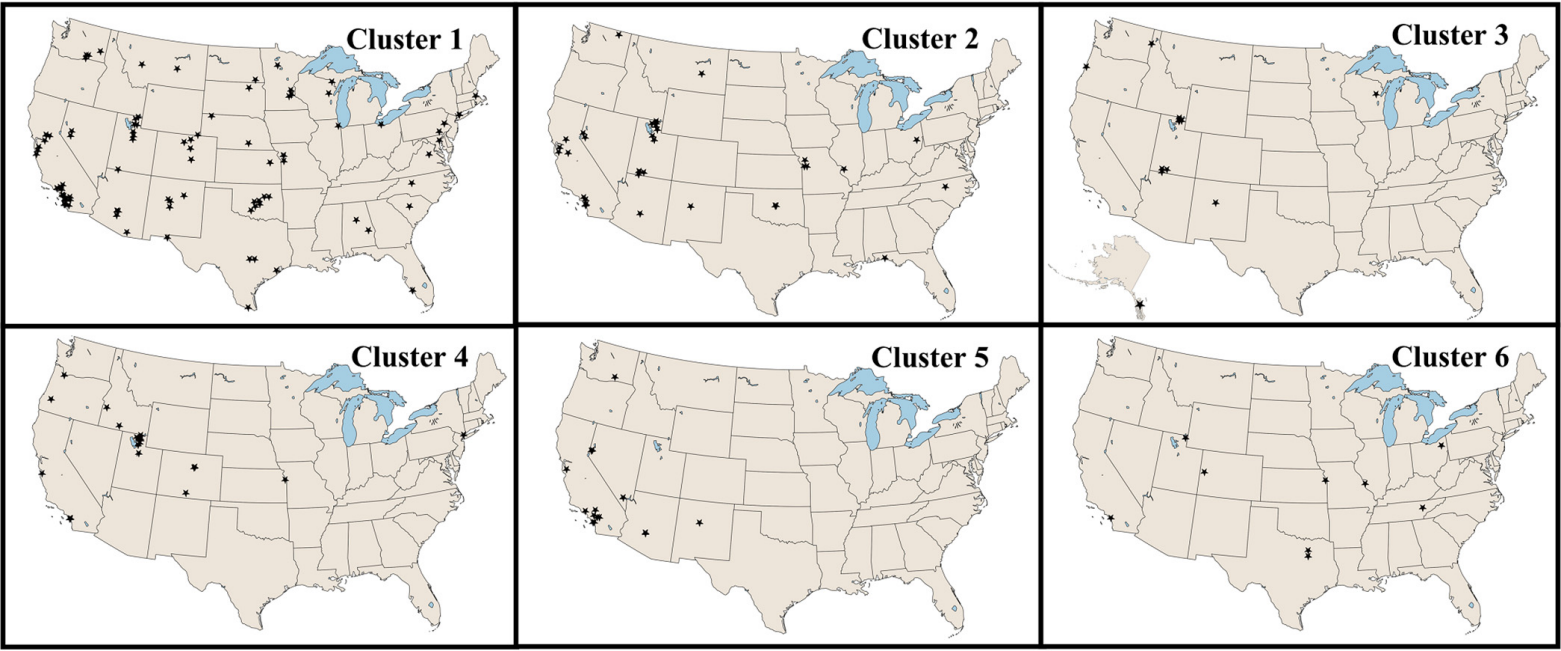
Chemical and geographical distribution of *Alternaria*. Shown is the geographical distribution of isolates by chemical cluster. Whereas clusters 1, 2, and 6 are well distributed throughout the study area, clusters 3, 4, and 5 occupy more limited ranges.

Considering the geographic scope of the collection, the genetic clade and chemical cluster data were evaluated to determine if their distributions might be limited to certain geographical regions (Fig. 2). Given the number of samples tested over such a large land mass, we are cautious in interpreting our results; however, we did note that cluster 5 was detected only in the far western portion of the United States. Additionally, clusters 3 and 4 were absent from the southeastern portion of the United States. Both observations served to fuel speculation that the occurrence of some *Alternaria* chemical features might be limited to circumscribed geographical ranges. Further investigation will be required to determine if these are veritable patterns or sampling artifacts.

### Chemical feature production among genetic clades.

Before proceeding, it is worth noting that in the comparisons presented here and in subsequent sections, the discussion could have been structured around evaluating *Alternaria* isolates according to ITS clades (genetics) or chemical features (metabolomics). Apart from clade V, our tests demonstrated rather strong agreement between the two models, which indicated that both clustering mechanisms worked well to organize data along seemingly natural divisions. Knowing that taxonomically driven strategies continue to play prominent roles in natural product collection efforts, we have opted to analyze the chemical diversity findings in the context of ITS clades (Fig. 1). However, we see no reason why a chemistry-centric grouping could not be used, and several examples of parallel tests based on chemical clusters are provided in the supplemental material.

**FIG 3:**
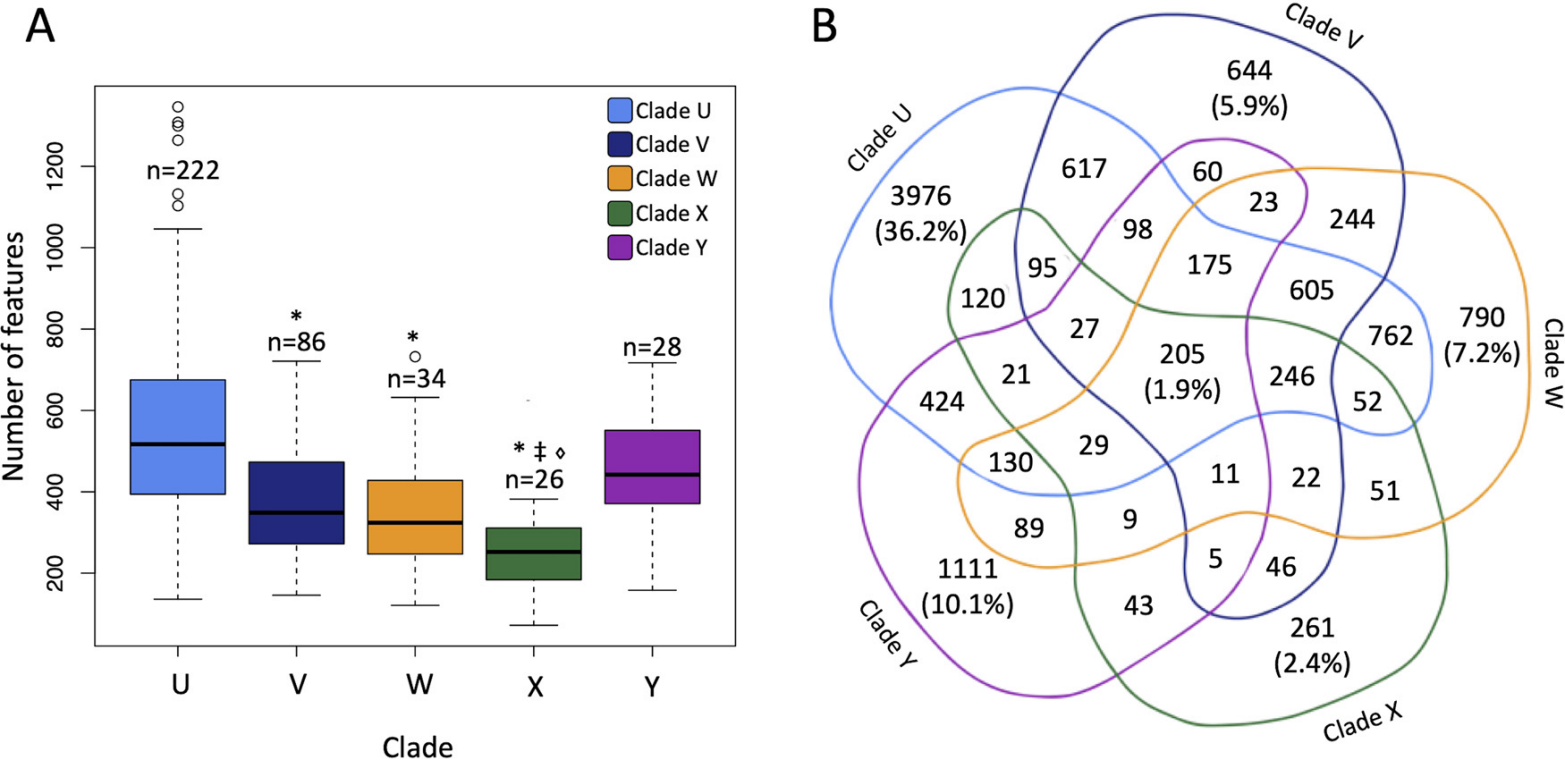
Summary of feature diversity in *Alternaria*. (A) Alpha diversity of genetic clades. The median numbers of chemical features differed significantly by clade. The asterisk indicates a statistically significant difference from clade U. The double dagger indicates a statistically significant difference from clade V. The diamond indicates a statistically significant difference from clade Y. (B) Venn diagram of features by clade.

Median numbers of detected chemical features differed significantly between ITS-based clades (*P* < 0.0001), with clades U and Y containing isolates that produced the greatest total numbers of chemical features (Fig. 3A). This observation held true (*P* < 0.0001) after subsampling of the clades to alleviate potential errors introduced due to sample size nonequivalence (Fig. S2A). Relatively few outliers were detected within the genetic clades, indicating high levels of consistency for the metabolic output of the isolates in each group. Clades V, W, and X were found to have significantly fewer features than clade U (Tukey’s honestly significant difference [HSD] of analysis of variance [ANOVA], *P* < 0.0001 in all cases), suggesting that clade U is chemically more diverse than the other clades.

Only 1.9% of features (205) were detected in all clades, comprising the core metabolome of the *Alternaria* isolates (Fig. 3B). While up to 40% of chemistry is shared between two or more clades, we found that the bulk of features were limited in occurrence to just a single clade. Progressing from the smallest to the largest number of clade-specific features, 2.4% of features (261) were found only in clade X, 5.9% of features (644) were present only in clade V, 7.2% of features (790) were detected only in clade W, 10.1% of features (1,111) were observed only in clade Y, and 36.2% of features (3,976) were identified only in clade U. These results demonstrate that high levels of chemical diversity exist even within the traditionally recognized boundaries that define *Alternaria*.

### Making informed library building decisions based on chemical feature diversity.

**FIG 4:**
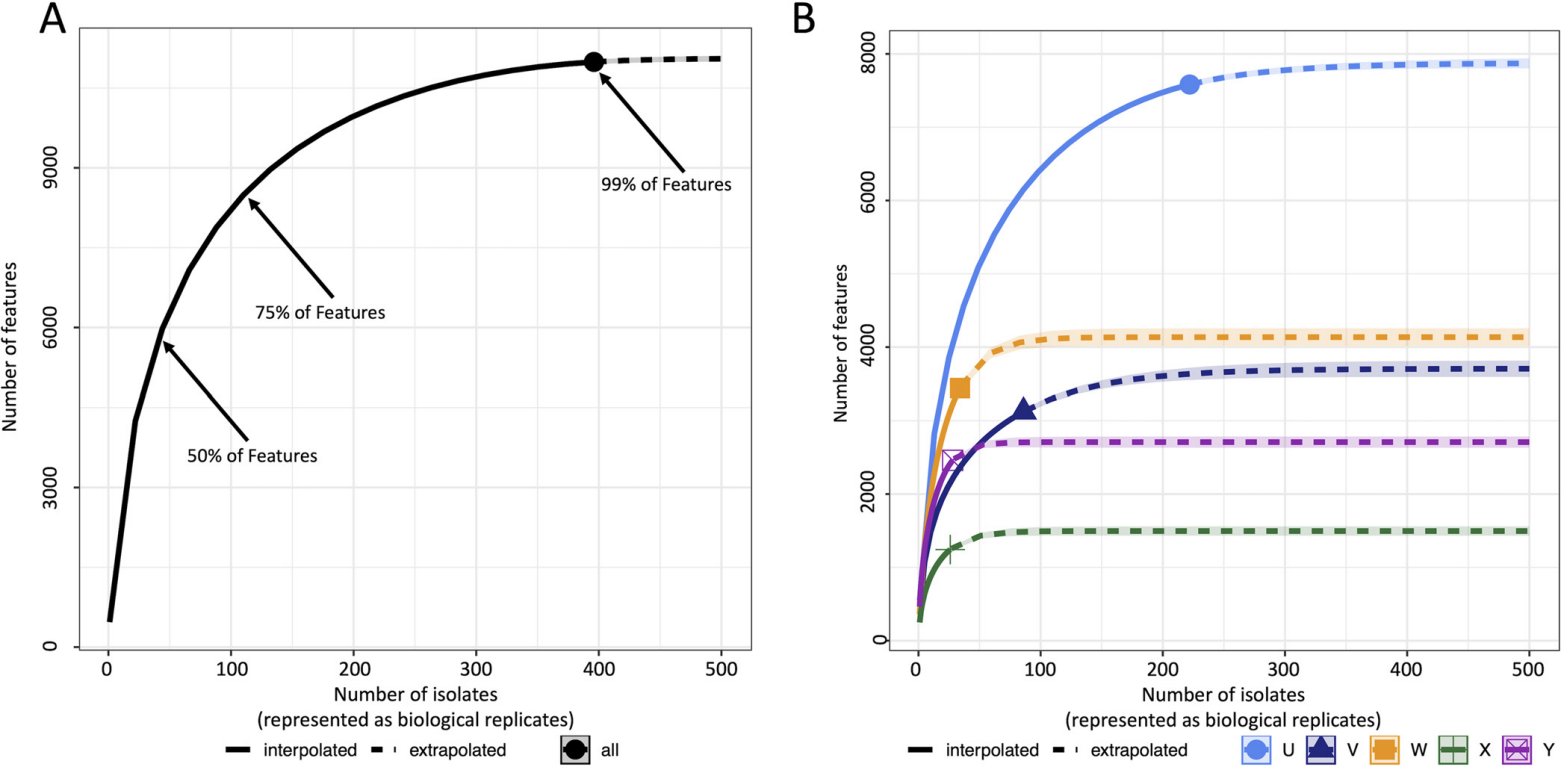
Chemical diversity curves and data extrapolation. (A) Rarefaction curve of chemical features within *Alternaria*. (B) Rarefaction curves for each ITS-based clade within *Alternaria*.

To monitor and better understand how feature diversity could be used to make informed decisions about constructing natural product libraries, feature accumulation curves were constructed from the metabolomics data (Fig. 4A). The results show that despite a large degree of ascribed taxonomic diversity in *Alternaria*, a surprisingly limited number of isolates are required to provide broad chemical coverage of the genus. Indeed, random sampling of the *Alternaria* data found that on average, a set consisting of as few as 23 isolates was expected to provide 50% of the total pool of *Alternaria* features. Expanding on these findings, randomly selected subsets consisting of 57, 104, 142, and 195 isolates were anticipated to provide 75%, 90%, 95%, and 99%, respectively, of *Alternaria* features (Fig. 4A). Thus, it was determined that feature accumulation data could serve as a useful tool for estimating levels of chemical feature coverage within taxonomic groups.

Whereas the genus-based amalgamation of feature data provided useful insights into the chemical diversity of *Alternaria*, a more granular exploration of feature accumulation results by subgenus clades has the potential to afford a complementary perspective for library design. Clade-based feature accumulation curves (Fig. 4B) showed that feature coverage levels of 99% were achievable in clades U (contained the most feature-rich isolates [Fig. 2A]) and X (contained the most feature-poor isolates [Fig. 2A]), with 170 and 51 total isolates, respectively. In contrast to the rank order of the median numbers of features per isolate, the point at which 99% feature saturation occurred followed a different pattern for clades V, W, and Y. Clade Y, which contained the second highest level of features per isolate (Fig. 2A), was found to require the lowest number of isolates (39 isolates) to achieve a level of 99% feature coverage. Clade V contained the third highest level of features per isolate (Fig. 2A), while also needing the second highest number of isolates (141 isolates) to achieve a level of 99% feature accumulation. These results are likely due to the presence of two chemical clusters being embedded in clade V. Clade W contained the second lowest number of features per isolate (Fig. 2A) but was predicted to require the third highest number of isolates (66 isolates) to achieve a level of 99% feature accumulation. Thus, feature accumulation curves utilizing ITS-based clades offer a useful method for identifying and monitoring genetically defined groups of organisms that are likely to require increased efforts (i.e., more isolates) to achieve prespecified levels of feature accumulation coverage. Related to these efforts, rarefaction curve slopes were plotted in relationship to the number of samples representing each clade (Fig. S4). The results of that analysis revealed that an inverse relationship existed between the slopes of interpolated rarefaction curves and the number of samples surveyed within a clade, supporting the idea that in this data set, the larger ITS-based clades tended to approach saturation of feature coverage.

10.1128/mSystems.00644-21.4 FIG S4 Relationship between size of clade and proximity to chemical saturation. In addition to using extrapolated rarefaction curves (Fig. 4A and B in the main text), the slope at the end of interpolated data in rarefaction curves reveals that larger clades have a lower slope, indicating that they are closer to saturation (slope = 0). Thus, the chemistry of larger clades is more fully described, and investigation of smaller clades may add more new features if sampled more extensively. Download FIG S4, TIF file, 0.7 MB. Copyright © 2021 Anderson et al. 2021 Anderson et al. https://creativecommons.org/licenses/by/4.0/ This content is distributed under the terms of the Creative Commons Attribution 4.0 International license.

### Probing of chemical scaffold distribution and diversity in *Alternaria*.

Whereas the analysis of chemical features offers a straightforward approach to comparing LC-MS data from different natural product sources, such results can be prone to misrepresenting underlying chemical diversity trends. Specifically, the output from natural product biosynthetic pathways tends to occur as assemblages of structurally related metabolites rather than as single products due to several factors related to the *in situ* formation of natural products, including substrate promiscuity, competing actions of multifarious tailoring enzymes, and more (47, 50, 51). Consolidating chemical features that share underlying structural similarities into groups referred to as scaffolds is one approach to account for this phenomenon. Molecular networking (52–55) is a method that has gained widespread use to build scaffold-level relationships in the field of natural products (56–59).

**FIG 5:**
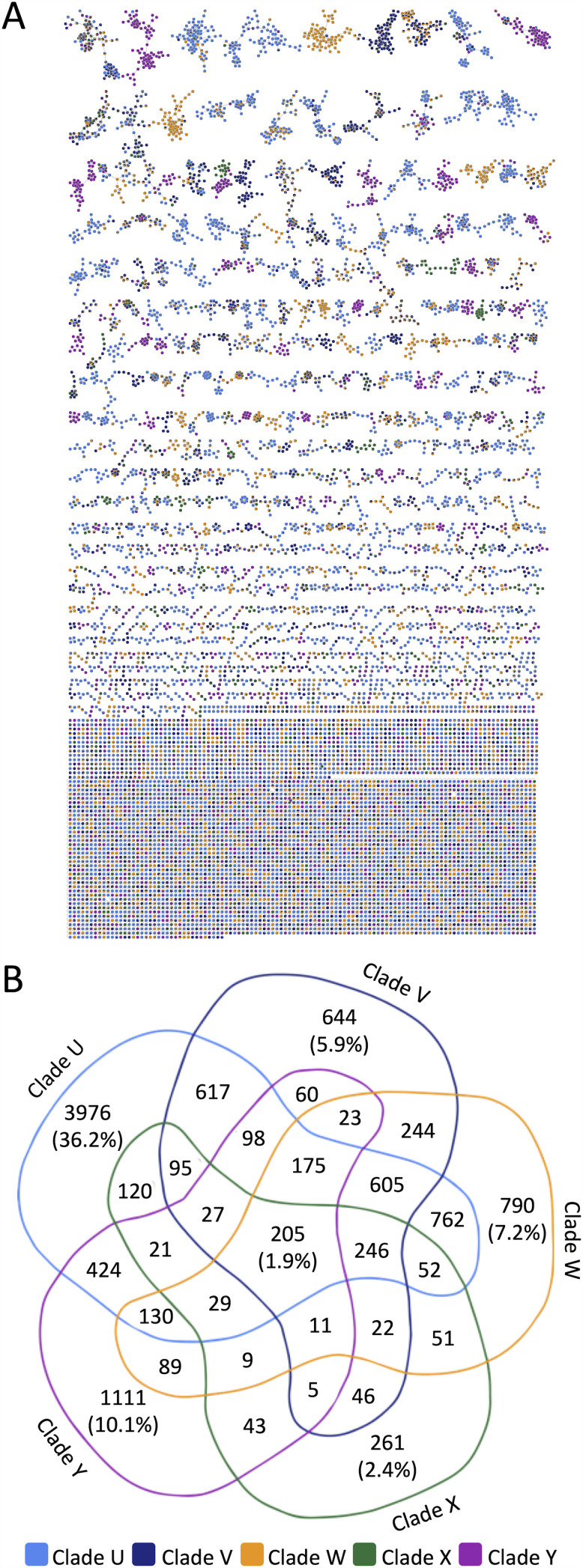
Scaffold diversity in *Alternaria*. (A) Results from molecular networking analysis constructed from LC-MS data reveal 5,754 subnetworks/scaffolds. Nodes are colored by clade. (B) Venn diagram illustrating chemical scaffolds by clade.

Using molecular networking to identify structurally related metabolites from *Alternaria*, the 10,991 molecular features were combined into 5,754 scaffolds (Fig. 5A). Upon removing singleton scaffolds (4,193) from the data set, 17.2% of the scaffolds (285) were found to be shared by all five ITS-based clades (Fig. 5B). These shared scaffolds represented the core metabolome of the *Alternaria* encountered in this study. We also found that 32.5% (539) of the nonsingleton scaffolds were detected in just a single clade. Clade U contained the largest number of unique chemical scaffolds (19.6% [326 unique scaffolds]), followed by clades Y (5.1% [84 unique scaffolds]), W (3.6% [59 unique scaffolds]), V (2.9% [48 unique scaffolds]), and X (1.3% [22 unique scaffolds]). The rank order of the scaffolds detected in a clade mirrored the respective levels of chemical features observed in each group (Fig. 2A). Thus, we speculate that the relative quantities of chemical features detected within taxa might serve as a surrogate measure for predicting their comparative levels of relative scaffold diversity, although further analysis will be necessary to explore this. These results also highlighted the need to differentiate scaffold versus feature diversity goals when establishing parameters for natural product library design, since 17.2% of scaffolds were found to be shared by all clades of *Alternaria*, but only 1.9% of features were shared by all clades. Furthermore, 61.7% of chemical features were found to be unique to a single clade, but this held true for only 32.5% of scaffolds, which indicates that many chemical scaffolds are conserved among *Alternaria* isolates.

### Applying clade and cluster data to assess progress toward goals for natural product library coverage.

**FIG 6:**
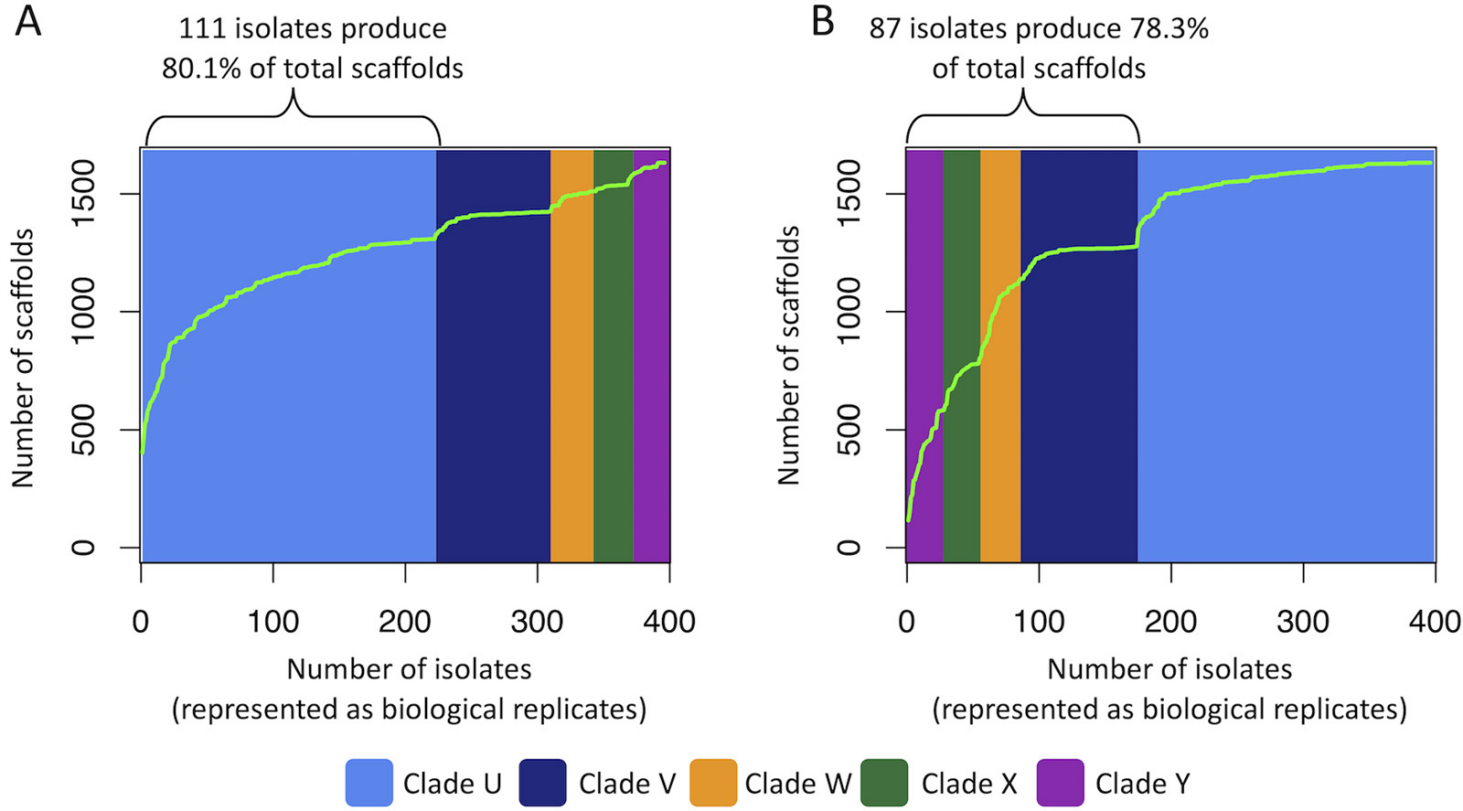
Visualization of scaffold accumulation models. (A) Scaffold accumulation curve generated starting with the most abundant clade (clade U) before adding isolates from the less abundant clades. (B) Scaffold accumulation curve generated by starting with less abundant clades (clades Y, X, W, and V) before introducing isolates from the most abundant clade.

Considering the entwined functions that phylogeny and chemistry have in natural product library development, we explored how less abundant taxa might contribute to the overall chemical diversity within a screening library. Such models could be useful for understanding how rigorous efforts to include less abundant taxa, or purposeful endeavors to exclude highly abundant groups of organisms, might impact the representation of chemical scaffolds in a collection. We first examined how forming a library by exclusively focusing on only the most abundant taxon, clade U, would affect the chemical diversity outcome of a collection (Fig. 6A and Fig. S6). The accumulation curves revealed that the 111 isolates in clade U could provide access to 80.1% of all *Alternaria* scaffolds, while the remaining, less abundant clades V, W, X, and Y added just 7.0%, 5.4%, 1.7%, and 5.7%, respectively, of additional chemical families (note that the order in which clades V, W, X, and Y were added was arbitrarily chosen). In contrast, when the scaffold accumulation data were examined with the focus placed on sampling just the less abundant taxa, it was found that the 87 isolates representing clades V, W, X, and Y afforded access to 78.3% of all scaffolds encountered from *Alternaria* (Fig. 6B). This result was unanticipated with near-equivalent percentages of unique scaffolds afforded via these contrasting approaches. We realize that most real-world library-building efforts are unlikely to engage in such restrictive collection practices; however, these results could have practical implications for cases in which searching out less abundant (i.e., rare taxa) or difficult-to-culture organisms may add undue cost or time to building a natural product drug screening library. Thus, modeling scaffold (or chemical feature) accumulation can help researchers focus on achieving desired levels of chemical coverage in natural product libraries, as well as monitoring whether collection efforts have led to oversaturation or undersampling of the theoretical chemical diversity within a given taxon.

10.1128/mSystems.00644-21.6 FIG S6 Adaption of collector’s curve for metabolomics analysis. In addition to rarefaction curves presented in Fig. 4A and B in the main text, the use of collector’s curve can shed additional light on the accumulation of chemistry. Collector’s curves differ from rarefaction curves in that they present the raw data as entered, while the rarefaction analysis creates a model for describing the smooth accumulation of diversity. Because these are raw data, the order of data can vastly change the shape and smoothness of the resulting curve. To illustrate the power of different arrangements of data on this method, the *Alternaria* data set was randomized 4 times in Microsoft Excel and scaffold accumulation curves were generated using the collector’s method in vegan. These curves were overlaid above. While the beginning and ending points of these curves are consistent (similar), the shapes between 1,000 and 1,400 are quite different. Download FIG S6, TIF file, 0.5 MB. Copyright © 2021 Anderson et al. 2021 Anderson et al. https://creativecommons.org/licenses/by/4.0/ This content is distributed under the terms of the Creative Commons Attribution 4.0 International license.

### Putting the pieces together to create natural product chemical collections.

It is our opinion that many efforts to construct natural product libraries have been based largely on opportunism and subjective reasoning rather than founded on data-driven goals and assessment. Whereas tremendous room exists to plot customized paths for building collections of secondary metabolites based on different parameters (e.g., genetic clades versus chemical clusters or features versus scaffolds), the best routes are likely to rely upon well-balanced sample collection strategies that combine appropriate amounts of chemical breadth in the resultant libraries. The purpose of our effort to measure natural product diversity was to afford researchers opportunities to establish library development goals and provide the means for assessing progress toward those targets. However, such goals should also be considered in the context of bioactive compound discovery, which in many ways is a heroic game of chance. To this point, we noted that within the *Alternaria* isolates studied, 17.9% of metabolite features were found in only a single isolate. Thus, overly stringent measures aimed at simply capturing only the core metabolome of genetic clades or chemical clusters risk missing outstanding pools of unique chemical matter that may prove critical for the success of a drug discovery program. We hope that these methods will help researchers set library building goals that are not only economical but also well poised to deliver the chemical matter needed to drive fruitful drug discovery operations.

## MATERIALS AND METHODS

### General sample selection and culture.

A cohort of 198 fungal isolates from the University of Oklahoma, Citizen Science Soil Collection, that had been identified as *Alternaria* were used in this study (Table S1). The map illustrating the sites where the isolates were obtained (Fig. 2) was generated in qGIS v3.10. The fungal isolates were identified based on BLASTN (60) comparisons of their ITS sequence data to the sequences of *Alternaria* type strains deposited in GenBank (60). When cultured on petri plates containing a modified potato dextrose agar, all isolates were determined to be consistent with the gross morphological features of *Alternaria* spp. For metabolomics experiments, the isolates were cultured for 3 weeks in duplicate, on a solid-state medium composed of Cheerios breakfast cereal supplemented with a 0.3% sucrose solution containing 0.005% chloramphenicol (61).

10.1128/mSystems.00644-21.8 TABLE S1 Source information for *Alternaria* isolates used in this analysis. Regions are NOAA regions based on the state from which each soil sample was submitted. The number of isolates in each group is indicated by a number in parentheses. Download Table S1, DOCX file, 0.04 MB. Copyright © 2021 Anderson et al. 2021 Anderson et al. https://creativecommons.org/licenses/by/4.0/ This content is distributed under the terms of the Creative Commons Attribution 4.0 International license.

### PCR and phylogenetic tree building.

Fungal cell lysates were prepared by removing fresh mycelium from each isolate and placing the samples in microcentrifuge tubes containing 200 μl of Tris-EDTA buffer (10 mM Tris-HCl, 1 mM disodium EDTA [pH 8.0]) and a 1:1 mixture of 1-mm and 0.5-mm zirconium oxide beads. Samples were homogenized using a BulletBlender (Next Advantage) set at maximum speed for 5 min. The 5.8S-ITS region was amplified by PCR using primers ITS1 (5′-TCCGTAGGTGAACCTGCGG-3′) and ITS4 (5′-TCCTCCGCTTATTGATATGC-3′) (62). Amplification and confirmation of PCR product formation were performed using a LightCycler 480 Instrument II (Roche) operated under the following conditions: 1 cycle of denaturation at 94°C for 2 min followed by 40 cycles of denaturation at 94°C for 1 min, annealing at 50°C for 1 min, and extension at 72°C for 1 min. Samples were submitted to Genewiz for Sanger sequencing with forward and reverse reads assembled using PhredPhrap (release 29) (minimum phred score: 50) (63, 64). Sequences were prepared for phylogenetic analysis using MEGA-X (65). ITS sequences for *Alternaria* type strains were obtained from the NCBI database (Table S2) (60). An outgroup consisting of five *Penicillium* spp. and five *Clonostachys* species isolates retrieved from the University of Oklahoma, Citizen Science Soil Collection, were used for tree rooting. Sequences were aligned using Clustal W in Mega X. Neighbor-joining tree analysis was carried out with 500 bootstraps using the Kimura2+G algorithm (65, 66).

### Metabolite sample preparation.

Samples for fungal metabolome analysis were prepared on an automated platform that combined both extraction and partitioning steps. Fungal cultures prepared in 16- by 100-mm borosilicate tubes were placed on a Tecan Freedom EVO platform and 3 ml of ethyl acetate was added to each sample. After extraction for 4 h, 3 ml of water was added to each tube to facilitate the partitioning process. Aliquots consisting of 2 ml of the upper ethyl acetate layers were transferred to deep-well 96-well plates. While the ethyl acetate was being removed from the samples *in vacuo*, the fungal culture tubes were each charged with an additional 3 ml of ethyl acetate to continue the partitioning process. The plates were returned to the liquid handler platform, at which point a second set of 2-ml aliquots of ethyl acetate was removed from the tubes and deposited into the deep-well 96-well plates. The organic solvent was removed *in vacuo* and the remaining organic residues were stored at −20°C for liquid chromatography-tandem mass spectrometry (LC-MS/MS) analysis.

### LC-MS/MS analysis.

Extracts were resuspended in 135 μl of 9:1 methanol-water spiked with 0.5 μM sulfadimethoxine, which served as an internal standard. Samples were analyzed on a Thermo Fisher Scientific Vanquish Flex Binary LC system, coupled to a Thermo Fisher Q Exactive Plus hybrid quadrupole-orbitrap mass spectrometer, using a C_18_ LC column (Kinetex, 50 by 2.1 mm, 1.7-μm particle size, 100-Å pore size; Phenomenex, Torrance, CA). The mobile phase consisted of LC-MS-grade acetonitrile and water (Fisher Optima; both eluents contained 0.1% formic acid). Sample elution was performed using a gradient system starting with 5% acetonitrile (held for 1 min), which was increased to 100% acetonitrile over 8 min and held at 100% acetonitrile for 2 min. Between samples, the eluent was returned to 5% acetonitrile over 30 s and held for 1 min before the next injection occurred. The column compartment and autosampler were held at 40°C and 10°C, respectively, for the duration of the analysis. Sample injection volumes of 5 μl were used, and samples were introduced in random order. Blanks and pooled quality control samples were interspersed throughout the analysis after every 12 samples. Electrospray conditions and data acquisition parameters are provided in Table S3 (part A).

10.1128/mSystems.00644-21.10 TABLE S3 Data acquisition and processing. (Part A) Data acquisition parameters for LC-MS/MS. (Part B) MZmine data processing parameters. (Part C) GNPS parameters. Download Table S3, DOCX file, 0.02 MB. Copyright © 2021 Anderson et al. 2021 Anderson et al. https://creativecommons.org/licenses/by/4.0/ This content is distributed under the terms of the Creative Commons Attribution 4.0 International license.

### Data processing and analyses.

Data were processed using MZmine v2.33 with the parameters provided in Table S3 (part B) (67). Data for the aligned peaks were exported from MZmine. All features identified as occurring in controls (blanks) and test samples were removed, and the remaining features were normalized to the total ion current (TIC) in the R statistical package. Principal-coordinate analysis (PCoA) and hierarchical clustering were performed on normalized tabulated data with QIIME1 (68) using a Bray-Curtis distance metric (69). The selection of 6 clusters was determined to be optimal based on a silhouette plot. Results were visualized using Emperor (70). Feature accumulation curves were made in vegan using binarized tabulated data (71), and plots were generated using a standard *x* axis representing the whole data set. Extrapolated rarefaction curves were generated in iNEXT with an endpoint of 500 duplicates (72, 73). Alpha diversity (observed chemical richness) was calculated using the Python package Scikit-Bio (version 0.2.0 [http://scikit-bio.org]) and analyzed using a one-way ANOVA and Tukey’s HSD test in R (74). To ensure that the differences in sample size did not skew analyses, balanced sets of randomly generated sample were analyzed for alpha diversity. Venn analyses were conducted using http://bioinformatics.psb.ugent.be/webtools/Venn/ and InteractiVenn (75). Global Natural Products Social Molecular Networking (GNPS) feature-based molecular networking was performed (52, 53) using output from MZmine2 (67) with the parameters described in Table S3 (part C).

### Data availability.

LC-MS/MS data were deposited in MassIVE under accession number MSV000083002. The feature-based molecular networking method is accessible at https://gnps.ucsd.edu/ProteoSAFe/status.jsp?task=f0608e9f1e0f4f3cb4d67bf16308e897. Sequencing data were deposited in GenBank under accession numbers MW729050 to MW729257. Codes for other analysis methods can be accessed on GitHub at https://github.com/NPDG/Alternaria.
