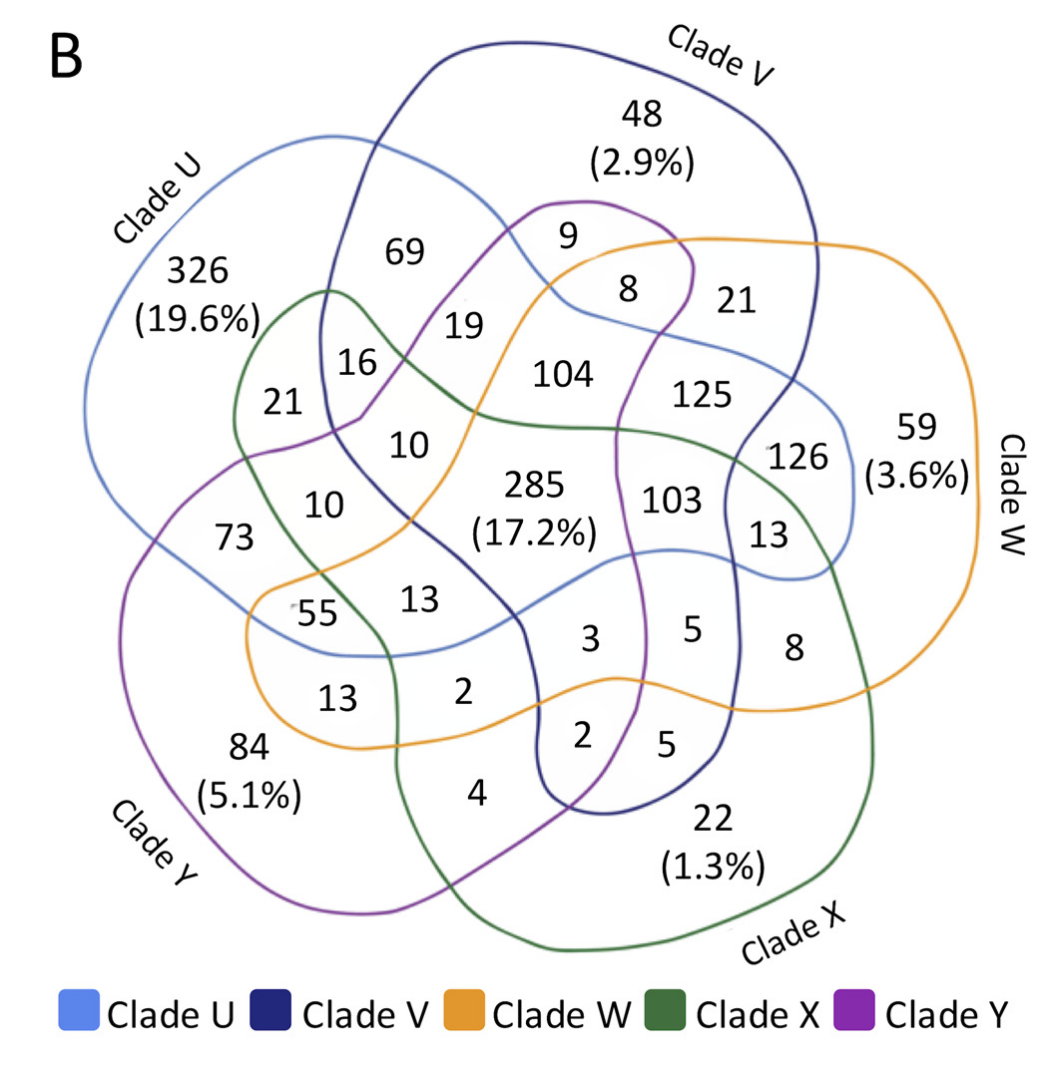# Correction for Anderson et al., “Building Natural Product Libraries Using Quantitative Clade-Based and Chemical Clustering Strategies”

**DOI:** 10.1128/msystems.01418-21

**Published:** 2021-12-14

**Authors:** Victoria M. Anderson, Karen L. Wendt, Fares Z. Najar, Laura-Isobel McCall, Robert H. Cichewicz

**Affiliations:** a Natural Products Discovery Group, University of Oklahoma, Norman, Oklahoma, USA; b Institute for Natural Products Applications and Research Technologies, University of Oklahoma, Norman, Oklahoma, USA; c Department of Chemistry and Biochemistry, University of Oklahoma, Norman, Oklahoma, USA; d Chemistry and Biochemistry Bioinformatics Core, University of Oklahoma, Norman, Oklahoma, USA; e Department of Microbiology and Plant Biology, University of Oklahoma, Norman, Oklahoma, USA; f Laboratories of Molecular Anthropology and Microbiome Research, University of Oklahoma, Norman, Oklahoma, USA

## AUTHOR CORRECTION

Volume 6, no. 5, e00644-21, 2021, https://doi.org/10.1128/mSystems.00644-21. Page 9: Fig. 5B should appear as shown below.

**Figure:**