# Supplementary material for: Building Natural Product Libraries Using Quantitative Clade-Based and Chemical Clustering Strategies
Source: mSystems. 2021 Oct 26;6(5):e00644-21. doi: 10.1128/mSystems.00644-21 (PMC8547436; doi:10.1128/mSystems.00644-21)
Supplement: TABLE S2 [file msystems.00644-21-st002.docx]

| **Number in tree** | **Type strain** | **Accession number** |
| --- | --- | --- |
| 1 | *Alternaria angustiovoidea* | MH861939 |
| 2 | *Alternaria cerealis* | NR_136117 |
| 3 | *Alternaria arborescens* | NR_135927 |
| 4 | *Alternaria daucifolii* | NR_137802 |
| 5 | *Alternaria alstroemeriae* | NR_163686 |
| 6 | *Alternaria destruens* | NR_137143 |
| 7 | *Alternaria tropica* | MH862449 |
| 8 | *Alternaria infectoria* | NR_131263 |
| 9 | *Alternaria dactylidicola* | NR_151852 |
| 10 | *Alternaria rosae* | NR_136017 |
| 11 | *Alternaria tellustris* | NR_135961 |
| 12 | *Alternaria molesta* | MH861376 |
| 13 | *Alternaria lolii* | NR_159632 |
| 14 | *Alternaria leptinellae* | NR_111866 |
| 15 | *Alternaria hungarica* | NR_135944 |
| 16 | *Alternaria hyacinthi* | NR_145168 |
| 17 | *Alternaria proteae* | NR_135930 |
| 18 | *Alternaria thalictrigena* | NR_135937 |
| 19 | *Alternaria zantedeschiae* | NR_160245 |
| 20 | *Alternaria sorghi* | NR_160246 |
| 21 | *Alternaria multiformis* | NR_077187 |
| 22 | *Alternaria terricola* | NR_103600 |
