# Supplementary material for: Building Natural Product Libraries Using Quantitative Clade-Based and Chemical Clustering Strategies
Source: mSystems. 2021 Oct 26;6(5):e00644-21. doi: 10.1128/mSystems.00644-21 (PMC8547436; doi:10.1128/mSystems.00644-21)
Supplement: TABLE S1 [file msystems.00644-21-st001.docx]

| Region | State | City | Sample ID | Full ID | Cryo ID |
| --- | --- | --- | --- | --- | --- |
| Alaska (1) | AK (1) | Douglas (1) | 106113 (1) | AK06113 RBM-3 | 200-A5 |
| Central (15) | IL (2) | O'Fallon (1) | 107958 (1) | IL07958 RBM+M-4 | 356-G10 |
|  |  | Oak Park (1) | 106098 (1)) | IL06098 RBM-1 | 330-C2 |
|  | MO (9) | Blue Springs (6) | 104924 (1) | MO04924 GVA-3 | 360-F7 |
|  |  |  | 104938 (1) | MO04938 RBM-3 | 307-A3 |
|  |  |  | 104941 (3) | MO04941 CZ-4 | 365-C8 |
|  |  |  |  | MO04941 PFA-8 | 365-D8 |
|  |  |  |  | MO04941 ZMA-1 | 365-D9 |
|  |  |  | 105221 (1) | MO05221 TV8-3 | 275-C3 |
|  |  | Lee's Summit (2) | 104933 (2) | MO04933 TV8-5 | 361-E4 |
|  |  |  |  | MO04933 SEA-1 | 361-G5 |
|  |  | Saint Louis (1) | 109829 (1) | MO09829 RBM-3 | 444-A2 |
|  | OH (3) | Dennison (1) | 12530 (1) | OH2530 CZSW-8 | 286-G9 |
|  |  | Lakeside Marblehead (1) | 12429 (1) | OH2429 TV8-4 | 197-B1 |
|  |  | Ravenna (1) | 13669 (1) | OH3669 PDA-2 | 354-B6 |
|  | TN (1) | Oak Ridge (1) | 108832 (1) | TN08832 RBM-2 | 411-C5 |
| East North Central (8) | MI (1) | Kingsford (1) | 106583 (1) | MI06583 RBM+M-4 | 244-G12 |
|  | MN (5) | Andover (1) | 101626 (1) | MN01626 TV8-2 | 175-F4 |
|  |  | Bemidji (1) | 11589 (1) | MN1589 TV8-2 | 253-G9 |
|  |  | Minneapolis (2) | 11708 (2) | MN1708 BSA-1 | 241-F8 |
|  |  |  |  | MN1708 BSA-2 | 241-F9 |
|  |  | Shakopee (1) | 15936 (1) | MN5936 TV8-2 | 170-A3 |
|  | WI (2) | Shawano (2) | 105148 (2) | WI05148 RBM-4 | 186-F11 |
|  |  |  |  | WI05148 RBM-1 | 186-F8 |
| Northeast (7) | CT (2) | Stamford (2) | 105458 (1) | CT05458 TV8-4 | 372-F4 |
|  |  |  | 105460 (1) | CT05460 TV8-3 | 319-E8 |
|  | MA (1) | Mattapan (1) | 102133 (1) | MA02133 RBM-4 | 185-D9 |
|  | MD (1) | Sparrows Point (1) | 108122 (1) | MD08122 BIA-4 | 282-E10 |
|  | NY (1) | Eastchester (1) | 101906 (1) | NY01906 GVA-1 | 335-F9 |
|  | PA (2) | Allentown (1) | 106591 (1) | PA06591 TV8+M-1 | 245-E10 |
|  |  | Lancaster (1) | 19696 (1) | PA9696 RBM-5 | 175-C5 |
| Northwest (12) | ID (3) | Post Falls (1) | 19935 (1) | ID9935 RBM-1 | 389-H2 |
|  |  | Star (1) | 107855 (1) | ID07855 RBM+M-1 | 356-F7 |
|  |  | Twin Falls (1) | 108003 (1) | ID08003 RBM+M-6 | 295-C11 |
|  | OR (3) | Portland (1) | 105493 (1) | OR05493 TV8-3 | 339-F8 |
|  |  | Roseburg (1) | 103007 (1) | OR03007 CZ-1 | 358-H1 |
|  |  | Yachats (1) | 105645 (1) | OR05645 TV8-2 | 236-G1 |
|  | WA (6) | Endicott (1) | 106230 (1) | WA06230 TV8+M-1 | 279-H6 |
|  |  | Republic (1) | 104059 (1) | WA04059 TV8-4 | 323-A8 |
|  |  | West Richland (4) | 106432 (4) | WA06432 TV8-2 | 239-G9 |
|  |  |  |  | WA06432 BIA-1 | 239-H3 |
|  |  |  |  | WA06432 BIA-6 | 239-H7 |
|  |  |  |  | WA06432 BIA-4 | 239-H8 |
| South (18) | KS (1) | Auburn (1) | 13211 (1) | KS3211 TV8-3 | 165-C11 |
|  | OK (9) | Marlow (1) | 106401 (1) | OK06401 TV8-1 | 347-D10 |
|  |  | Mounds (2) | 105088 (2) | OK05088 RBM-3 | 342-G4 |
|  |  |  |  | OK05088 RBM-4 | 342-G5 |
|  |  | Oklahoma City (5) | 102375 (1) | OK02375 RBM-4 | 333-E11 |
|  |  |  | 104301 (3) | OK04301 TV8-7 | 177-E1 |
|  |  |  |  | OK04301 RBM-4 | 177-E5 |
|  |  |  |  | OK04301 RBM-5 | 177-E6 |
|  |  |  | 107080 (1) | OK07080 RBM-4 | 310-G10 |
|  |  | Tecumseh (1) | 10626 (1) | Tucker BIA-1 | 154-A4 |
|  | TX (8) | Alvin (1) | 104415 (1) | TX04415 CIT-4 | 349-G2 |
|  |  | Austin (2) | 106180 (2) | TX06180 RBM-1 | 239-D10 |
|  |  |  |  | TX06180 BIA-3 | 239-D12 |
|  |  | Dallas (2) | 103115 (1) | TX03115 RBM-2 | 325-E11 |
|  |  |  | 103143 (1) | TX03143 PFA-2 | 369-C2 |
|  |  | El Paso (2) | 15878 (1) | TX5878 RBM-4 | 145-B9 |
|  |  |  | 18357 (1) | TX8357 RBM-1 | 133-H4 |
|  |  | Weslaco (1) | 19737 (1) | TX9737 BIA-2 | 380-A8 |
| Southeast (8) | AL (2) | Birmingham (1) | 12730 (1) | AL2730 BIA-2 | 258-B8 |
|  |  | Tuskegee (1) | 106505 (1) | AL06505 TV8+M-2 | 337-B2 |
|  | FL (2) | Cape Coral (1) | 15539 (1) | FL5539 TV8-3 | 201-E4 |
|  |  | Niceville (1) | 105029 (1) | FL05029 TV8-1 | 331-B9 |
|  | NC (2) | Chapel Hill (1) | 107859 (1) | NC07859 RBM-3 | 431-A2 |
|  |  | Clemmons (1) | 14376 (1) | NC4376 RBM-5 | 404-B10 |
|  | SC (1) | Columbia (1) | 15920 (1) | SC5920 TV8-1 | 101-H7 |
|  | VA (1) | Unionville (1) | 19846 (1) | VA9846 TV8-2 | 175-A7 |
| Southwest (61) | AZ (8) | Phoenix (5) | 101714 (1) | AZ01714 RBM+M-5 | 333-A8 |
|  |  |  | 105667 (3) | AZ05667 TV8-3 | 339-A11 |
|  |  |  |  | AZ05667 RBM-7 | 340-B7 |
|  |  |  |  | AZ05667 TV8-2 | 346-A6 |
|  |  |  | 106110 (1) | AZ06110 RBM-1 | 310-A12 |
|  |  | Scottsdale (1) | 105966 (1) | AZ05966 RBM-1 | 306-B5 |
|  |  | Sonoita (1) | 107035 (1) | AZ07035 RBM+M-1 | 337-A5 |
|  |  | Tempe (1) | 100157 (1) | AZ00157 RBM-1 | 199-A7 |
|  | CO (8) | Alamosa (1) | 103739 (1) | CO03739 RBM-5 | 315-H8 |
|  |  | Arvada (1) | 17626 (1) | CO7626 TV8-3 | 149-A5 |
|  |  | Colorado Springs (1) | 106011 (1) | CO06011 RBM-4 | 360-C1 |
|  |  | Denver (1) | 15842 (1) | CO5842 RBM-1 | 136-B12 |
|  |  | Fort Collins (1) | 11330 (1) | CO1330 RBM-3 | 147-B2 |
|  |  | Golden (1) | 15585 (1) | CO5585 RBM+M-4 | 308-A12 |
|  |  | Grand Junction (1) | 108658 (1) | CO08658 RBM-4 | 421-C11 |
|  |  | Grand Lake (1) | 105854 (1) | CO05854 BIA-1 | 238-B1 |
|  | NM (7) | Albuquerque (6) | 16512 (2) | NM6512 RBM-2 | 134-D5 |
|  |  |  |  | NM6512 RBM-1 | 147-C6 |
|  |  |  | 16572 (1) | NM6572 TV8-3 | 139-F1 |
|  |  |  | 16579 (1) | NM6579 TV8-1 | 139-F2 |
|  |  |  | 101594 (1) | NM01594 RBM-4 | 179-A9 |
|  |  |  | 104076 (1) | NM04076 SULF-2 | 390-E1 |
|  |  | Serafina (1) | 13634 (1) | NY3634 TV8-2 | 159-E5 |
|  | UT (38) | Hyde Park (2) | 107209 (2) | UT07209 TV8-3 | 269-E10 |
|  |  |  |  | UT07209 TV8-1 | 269-E9 |
|  |  | Layton (1) | 107902 (1) | UT07902 TV8+M-1 | 296-H5 |
|  |  | Lindon (1) | 107814 (1) | UT07814 RBM+M-1 | 356-B9 |
|  |  | Logan (15) | 106978 (1) | UT06978 RBM-1 | 264-D2 |
|  |  |  | 107120 (1) | UT07120 TV8-3 | 267-F12 |
|  |  |  | 107129 (1) | UT07129 RBM-2 | 269-D2 |
|  |  |  | 107162 (1) | UT07162 TV8-3 | 267-A5 |
|  |  |  | 107164 (1) | UT07164 TV8-1 | 267-B12 |
|  |  |  | 107193 (1) | UT07193 TV8-6 | 264-C2 |
|  |  |  | 107195 (1) | UT07195 RBM-2 | 268-F1 |
|  |  |  | 107285 (1) | UT07285 TV8-2 | 355-C2 |
|  |  |  | 107825 (3) | UT07825 RBM-3 | 334-A8 |
|  |  |  |  | UT07825 TV8-6 | 355-B5 |
|  |  |  |  | UT07825 BIA-2 | 355-B6 |
|  |  |  | 109117 (2) | UT09117 RBM-5 | 386-A11 |
|  |  |  |  | UT09117 SULF-4 | 386-A2 |
|  |  |  | 109210 (1) | UT09210 TV8-3 | 383-G3 |
|  |  |  | 109630 (1) | UT09630 RBM-4 | 444-A9 |
|  |  | Orderville (9) | 16905 (2) | UT6905 TV8-4 | 194-H4 |
|  |  |  |  | UT6905 RBM-4 | 197-D9 |
|  |  |  | 16917 (1) | UT6917 RBM-30 | 197-E10 |
|  |  |  | 16918 (1) | UT6918 RBM-1 | 170-F4 |
|  |  |  | 16921 (1) | UT6921 RBM-1 | 164-D9 |
|  |  |  | 16925 (2) | UT6925 TV8-1 | 169-E8 |
|  |  |  |  | UT6925 TV8-2 | 169-E9 |
|  |  |  | 16926 (1) | UT6926 RBM-1 | 157-H2 |
|  |  |  | 16927 (1) | UT6927 RBM-1 | 190-D4 |
|  |  | Orem (1) | 101299 (1) | UT01299 RBM-5 | 166-G4 |
|  |  | Paradise (2) | 109111 (2) | UT09111 RBM-3 | 386-F1 |
|  |  |  |  | UT09111 RBM-4 | 386-F2 |
|  |  | Payson (1) | 102892 (1) | UT02892 RBM-3 | 331-G7 |
|  |  | Provo (1) | 106863 (1) | UT06863 RBM+M-2 | 247-G9 |
|  |  | Sandy (1) | 12290 (1) | UT2290 RBM-2 | 170-E12 |
|  |  | Tremonton (2) | 107838 (2) | UT07838 RBM+M-3 | 282-B3 |
|  |  |  |  | UT07838 TV8+M-2 | 282-B6 |
|  |  | Wellsville (1) | 108880 (1) | UT08880 RBM-4 | 385-D2 |
|  |  | West Jordan (1) | 106991 (1) | UT06991 SULF-3 | 362-C3 |
| West (58) | CA (50) | Canyon Country (2) | 104365 (2) | CA04365 RBM-7 | 272-A11 |
|  |  |  |  | CA04365 RBM-3 | 272-A8 |
|  |  | Capistrano Beach (3) | 106897 (1) | CA06897 TV8-1 | 253-D6 |
|  |  |  | 106910 (2) | CA06910 TV8-2 | 260-D9 |
|  |  |  |  | CA06910 RBM-5 | 262-B7 |
|  |  | Dana Point (3) | 106912 (1) | CA06912 TV8-5 | 260-D5 |
|  |  |  | 107639 (1) | CA07639 RBM-1 | 387-E1 |
|  |  |  | 107649 (1) | CA07649 BIA-1 | 390-D4 |
|  |  | Dublin (2) | 19212 (1) | CA9212 RBM-2 | 152-B2 |
|  |  |  | 19443 (1) | CA9443 RBM-1 | 166-H2 |
|  |  | Garden Grove (1) | 100516 (1) | CA00516 RBM-2 | 167-A11 |
|  |  | La Puente (1) | 19633 (1) | CA9633 RBM-1 | 151-A8 |
|  |  | Ladera Ranch (5) | 106893 (1) | CA06893 RBM-1 | 260-C1 |
|  |  |  | 106898 (1) | CA06898 RBM-4 | 268-B1 |
|  |  |  | 106905 (1) | CA06905 RBM-2 | 266-C12 |
|  |  |  | 106924 (1) | CA06924 TV8-1 | 267-G2 |
|  |  |  | 107564 (1) | CA07564 RBM-1 | 387-C3 |
|  |  | Los Alamitos (1) | 106077 (1) | CA06077 TV8-4 | 380-H8 |
|  |  | Marina (1) | 12503 (1) | CA2503 RBM-2 | 178-D1 |
|  |  | Perris (1) | 105759 (1) | CA05759 RBM+M-1 | 280-A8 |
|  |  | Pomona (2) | 103709 (2) | CA03709 SULF-5 | 382-E5 |
|  |  |  |  | CA03709 SULF-6 | 382-E6 |
|  |  | Redlands (1) | 105688 (1) | CA05688 RBM-4 | 347-B1 |
|  |  | Rio Linda (2) | 16630 (1) | CA6630 RBM-5 | 168-E2 |
|  |  |  | 105902 (1) | CA05902 RBM+M-3 | 308-G8 |
|  |  | Riverside (1) | 100382 (1) | CA00382 CEA-1 | 365-G7 |
|  |  | Sacramento (3) | 102293 (3) | CA02293 RBM-3 | 173-C6 |
|  |  |  |  | CA02293 RBM-4 | 180-B10 |
|  |  |  |  | CA02293 RBM-2 | 180-B9 |
|  |  | San Clemente (2) | 106904 (2) | CA06904 RBM-3 | 263-C1 |
|  |  |  |  | CA06904 TV8-1 | 263-C2 |
|  |  | San Diego (1) | 100380 (1) | CA00380 RBM-3 | 157-F2 |
|  |  | San Jose (5) | 16130 (2) | CA6130 CGA-1 | 153-E12 |
|  |  |  |  | CA6130 BIA-2 | 155-E5 |
|  |  |  | 105322 (1) | CA05322 PFA-3 | 348-B5 |
|  |  |  | 106256 (2) | CA06256 RBM-5 | 280-B9 |
|  |  |  |  | CA06256 TV8-3 | 307-C11 |
|  |  | San Juan Capistrano (5) | 106890 (1) | CA06890 TV8-1 | 387-A9 |
|  |  |  | 106919 (1) | CA06919 RBM-3 | 268-A6 |
|  |  |  | 106930 (1) | CA06930 RBM-3 | 266-H2 |
|  |  |  | 106932 (1) | CA06932 RBM-1 | 268-A8 |
|  |  |  | 107634 (1) | CA07634 TV8-1 | 386-G8 |
|  |  | Santa Ana (1) | 105894 (1) | CA05894 RBM-2 | 352-A7 |
|  |  | Simi Valley (5) | 100535 (5) | CA00535 MEA-4 | 359-A3 |
|  |  |  |  | CA00535 BFA-2 | 359-A7 |
|  |  |  |  | CA00535 RBM-3 | 359-B7 |
|  |  |  |  | CA00535 RBM-5 | 359-B9 |
|  |  |  |  | CA00535 CZSW-2 | 359-D5 |
|  |  | Turlock (1) | 107860 (1) | CA07860 TV8-2 | 421-A1 |
|  |  | Yorba Linda (1) | 100742 (1) | CA00742 TV8-1 | 201-B8 |
|  | NV (8) | Dayton (1) | 17690 (1) | NV7690 TV8-2 | 158-H6 |
|  |  | Fallon (2) | 107695 (2) | NV07695 RBM-3 | 316-E1 |
|  |  |  |  | NV07695 RBM-4 | 316-E2 |
|  |  | Las Vegas (2) | 102048 (1) | NV02048 CIT-1 | 439-C7 |
|  |  |  | 108352 (1) | NV08352 RBM-5 | 411-C10 |
|  |  | Reno (2) | 107748 (2) | NV07748 CIT-1 | 423-E8 |
|  |  |  |  | NV07748 SULF-5 | 423-F5 |
|  |  | Sparks (1) | 103768 (1) | NV03768 TV8-1 | 326-C4 |
| West North Central (10) | MT (3) | Helena (1) | 13034 (1) | MT3034 RBM-4 | 148-E3 |
|  |  | Melstone (2) | 106089 (2) | MT06089 RBM-3 | 353-E9 |
|  |  |  |  | MT06089 RBM-1 | 366-D1 |
|  | ND (1) | Gwinner (1) | 101000 (1) | ND01000 RBM-6 | 374-F7 |
|  | NE (2) | Chadron (1) | 101209 (1) | NE01209 TV8-7 | 185-F7 |
|  |  | Hastings (1) | 104278 (1) | NE04278 TV8-8 | 347-D12 |
|  | SD (1) | Aberdeen (1) | 16748 (1) | SD6748 TV8-3 | 183-H4 |
|  | WY (3) | Carpenter (1) | 14702 (1) | WY4702 RBM-3 | 396-C10 |
|  |  | Otto (2) | 107136 (2) | WY07136 RBM-2 | 269-B2 |
|  |  |  |  | WY07136 RBM-4 | 269-B4 |
