# Supplementary material for: Building Natural Product Libraries Using Quantitative Clade-Based and Chemical Clustering Strategies
Source: mSystems. 2021 Oct 26;6(5):e00644-21. doi: 10.1128/mSystems.00644-21 (PMC8547436; doi:10.1128/mSystems.00644-21)
Supplement: TABLE S3 [file msystems.00644-21-st003.docx]

Part A

| **Parameter** | **Value** |
| --- | --- |
| Data acquisition mode | positive |
| Scan range | 100-1500 m/z |
| MS1 Resolution | 35,000 |
| MS 2 Resolution | 17,500 |
| Top N | 5 |
| sheath gas | 35 L/min |
| auxiliary gas | 10 L/min |
| sweep gas | 0 L/min |
| auxiliary gas temperature | 350 C |
| spray voltage | 3.8 kV |
| S-lens RF | 50 V |
| capillary temperature | 320 C |
| Maximum injection time (MS1 & MS2) | 100 Ms |
| MS1 AGC target | 1E6 |
| MS2 AGC target | 5E5 |
| Isolation window | 2 *m/z* |
| Normalized collision energy increments | 20%, 30%, 40% |
| MS2 dynamic exclusion | 10 s |
| Apex trigger | 2-8 s |
| Exclude | Unassigned charges |

Part B

| **Process** | **Parameter** | **Value** |
| --- | --- | --- |
| Mass Detection | MS1 Noise Level | 4.0E5 |
|  | MS2 Noise Level | 6.00E+03 |
|  | Mass Detector | Centroid |
| Chromatogram Builder | Minimum Time Span (min) | 0.01 |
|  | Minimum Height | 1E7 |
|  | m/z tolerance (ppm) | 10 |
| Chromatogram Deconvolution: LOCAL MINIMA algorithm | Chromatographic threshold | 20 |
|  | Search minimum in RT range (min) | .08 |
|  | Minimum relative height | 26 |
|  | Minimum absolute height | 1E7 |
|  | Min ratio of peak top/edge | 1.19 |
|  | Peak duration range (min) | 0.01-1.00 |
|  | *m/z* Range for MS2 Scan Pairing (Da) | 0.01 |
|  | RT Range for MS2 Scan Pairing (min) | 0.1 |
| Isotopic Peak Grouper | Retention Time Tolerance (min) | 0.1 |
|  | *m/z* tolerance (ppm) | 10 |
|  | Monotonic Shape | Yes |
|  | Maximum Charge | 3 |
|  | Representative isotope | Lowest *m/z* |
| Join aligner | *m/z* tolerance (ppm) | 15 |
|  | *m/z* to RT weight | 1-1 |
|  | Retention Time Tolerance (min) | 0.25 |
| Row filtering | Retention Time | 0.20-12 min |
|  | Keep only peaks with MS2 scan | Enabled |
|  | Minimum peaks in a row | 2 (for duplicates) |

Part C

| **Parameter** | **Value** |
| --- | --- |
| MS/MS fragment ions filtering | +/- 17 Da of the precursor m/z |
| MS/MS spectra were window filtered | 6 fragment ions in the +/- 50 Da window |
| precursor ion mass tolerance | 0.02 Da |
| MS/MS fragment ion tolerance | 0.02 Da |
| cosine score | ≥0.7 |
| Minimum matched peaks | 4 |
| edges between two nodes | 10 most similar nodes |
| maximum size of a molecular family | 100 |
| analogue search mode | enabled |
| MS/MS spectra | 200.0 |
| matches kept between network spectra and library spectra |  |
| cosine score | ≥0.7 |
| Minimum matched peaks | 4 |
